# Increased risk of stroke in patients with diffuse idiopathic skeletal hyperostosis: a nationwide population-based cohort study

**DOI:** 10.1038/s41598-021-00798-2

**Published:** 2021-11-01

**Authors:** Yuan-Yang Cheng, Ching-Heng Lin, Po-Yi Tsai, Yi-Huei Chen, Shih-Yi Lin, Shin-Tsu Chang

**Affiliations:** 1 Department of Physical Medicine and Rehabilitation, Taichung Veterans General Hospital, No. 1650, Taiwan Boulevard Sect. 4, Taichung, 407219 Taiwan; 2 School of Medicine, National Yang Ming Chiao Tung University, Hsinchu, Taiwan; 3 Center for Geriatrics and Gerontology, Taichung Veterans General Hospital, Taichung, Taiwan; 4 Department of Medical Research, Taichung Veterans General Hospital, Taichung, Taiwan; 5 Department of Physical Medicine and Rehabilitation, Taipei Veterans General Hospital, Taipei, Taiwan; 6 Department of Physical Medicine and Rehabilitation, Kaohsiung Veterans General Hospital, No. 386, Dazhong 1st Rd., Zuoying Dist., Kaohsiung, 813414 Taiwan; 7 Department of Physical Medicine and Rehabilitation, School of Medicine, Tri-Service General Hospital, National Defense Medical Center, No. 161, Section 6, Minquan East Road, Neihu District, Taipei, 114201 Taiwan

**Keywords:** Neurology, Rheumatology, Risk factors

## Abstract

Diffuse idiopathic skeletal hyperostosis (DISH) is frequently an incidental finding during X-ray examination. Although it has been shown to be associated with several chronic diseases, the hazard of cerebrovascular disease has seldom been explored. Our study aimed at determining the risk of stroke conferred by DISH, which is a retrospective cohort study adopting the largest medical database in Taiwan. Patients with a diagnosis of DISH at least three times from 2005 to 2010 were identified as the study group, and those in the control group were selected by matching age and gender. Patients were followed up until the end of 2015 to trace the incidence of stroke. Cox regression analysis was performed to compute the hazard ratio of stroke. Among the included 5300 patients, 1060 had a diagnosis of DISH. Significantly higher prevalence rates of stroke, hypertension, diabetes, and hyperlipidemia were noted in these patients. Overall, DISH conferred a 1.68 times higher risk of developing stroke. The significantly higher hazard ratio could be identified in both genders whether hypertension existed or not. Even in those without comorbidities, DISH still conferred a significantly higher risk of cerebrovascular disease in the future, which should never be ignored when encountered during clinical practice.

## Introduction

Diffuse idiopathic skeletal hyperostosis (DISH), an idiopathic rheumatologic abnormality, is characterized by exuberant ossification along ligaments throughout the body, most notably at the anterior longitudinal ligament of the spine^1^. This phenomenon was first observed in 1950^2^, and was later defined by Resnick^3^ as the presence of continuous calcification and ossification along the anterolateral flank of at least 4 contiguous vertebral bodies without decreased intervertebral height or erosion and sclerosis of the sacroiliac joints. Despite being a relatively common disease, the etiology has not been fully clarified^4^. Some authors have postulated that its pathogenic pathway is closed related to ossification of the posterior longitudinal ligament^5^, and both excess growth factors that may transform mesenchymal cells into osteoblasts^6^ and reduced activity of inhibitors of bone-promoting peptides^7^ have been proposed to be involved in the etiology of DISH.

Despite having a prominent radiological feature, DISH is usually asymptomatic. The most frequently encountered symptoms are back pain and stiffness^8^. While conflicting data exist with regard to the prevalence of pain^9^, difficulty in bending forward and impaired physical function are considered to be the most common problems in DISH patients^10^. Furthermore, because hyperostosis is not limited to the spine, extraspinal entheseal ossifications and atypical osteoarthritic change of peripheral joints are also frequently reported^11^. In addition, dysphagia and airway obstruction can also develop due to abundant bone located anterior to the vertebral bodies in the cervical spine displacing the trachea and esophagus^12^. Finally, in traumatic cases involving the neck region, the risks of spinal fracture and neurological injury were reported to be much higher in patients with DISH^13,14^.

Aside from the above clinical problems, the value of the diagnosis of DISH may lie in its close relationship with several vascular risk factors. In the literature, it has been shown that patients with DISH are prone to have metabolic syndrome and have a higher risk of coronary heart disease^15–17^. Conversely, patients with advanced cardiovascular diseases were also found to have a higher prevalence of DISH^18^. Although the causal relationship and the underlying mechanism have still not been completely elucidated, the close relationship between heart diseases and DISH should never be underestimated. While a number of studies have been conducted on the association between DISH and coronary heart disease, there are few data on the relationship of this bone disorder with cerebrovascular diseases. Only one study revealed a higher incidence of cerebral infarction and stenosis or occlusion of a major cerebral artery in patients with DISH^19^. Stroke is one of the leading causes of death worldwide^20^, and the impairment of physical function and self-care ability, as well as the burden placed on healthcare resources, that arise as a consequence of this disease remains a major public health issue. Therefore, in this retrospective study, we aimed to determine whether DISH patients had a higher risk of stroke, and to evaluate the hazard ratios of stroke in different groups of patients based on age, gender, and comorbidities.

## Results

The distribution of our study subjects categorized by age, gender, and comorbidities are shown in Table 1. Because the subjects without DISH were selected from the database by matching with DISH patients by age and gender, no significant differences were noted in age and gender between the two groups. However, stroke, hypertension, diabetes mellitus, and hyperlipidemia were significantly more prevalent in patients with DISH (*p* < 0.05), though there was no difference in prevalence of chronic kidney disease.

**Table 1:** Clinical characteristics of study subjects with and without diffuse idiopathic skeletal hyperostosis.

Variables	Total (n = 5300)		Without DISH (n = 4240)		With DISH (n = 1060)		*P* value
	n	(%)	n	(%)	n	(%)	
**Age, years**							0.260
50–60	1935	(36.5)	1548	(36.5)	387	(36.5)	
60–70	1575	(29.7)	1241	(29.3)	334	(31.5)	
≥ 70	1790	(33.8)	1451	(34.2)	339	(32.0)	
**Gender**							1.000
Female	2955	(55.8)	2364	(55.8)	591	(55.8)	
Male	2345	(44.2)	1876	(44.2)	469	(44.2)	
**Stroke**							< 0.001*
No	4596	(86.7)	3758	(88.6)	838	(79.1)	
Yes	704	(13.3)	482	(11.4)	222	(20.9)	
**Hypertension**							< 0.001*
No	3656	(69.0)	3062	(72.2)	594	(56.0)	
Yes	1644	(31.0)	1178	(27.8)	466	(44.0)	
**Diabetes mellitus**							0.001*
No	4652	(87.8)	3753	(88.5)	899	(84.8)	
Yes	648	(12.2)	487	(11.5)	161	(15.2)	
**Hyperlipidemia**							< 0.001*
No	4541	(85.7)	3717	(87.7)	824	(77.7)	
Yes	759	(14.3)	523	(12.3)	236	(22.3)	
**Chronic kidney disease**							0.943
No	5251	(99.1)	4201	(99.1)	1050	(99.1)	
Yes	49	(0.9)	39	(0.9)	10	(0.9)	

*DISH* diffuse idiopathic skeletal hyperostosis.

*p < 0.05.

All of our study subjects were then traced to determine the incidence of stroke until December 31st, 2015. The time interval between the diagnosis of DISH and stroke onset was 3.5 ± 2.6 years, while the interval between the index date and stroke onset was 3.9 ± 2.6 years in patients without DISH. In order to identify the hazard of stroke development according to the variables that were evaluated, Cox regression analysis was performed. As shown in Table 2, a significantly higher hazard ratio was noted in patients who were older, and in those with DISH, hypertension, or diabetes mellitus. The adjusted hazard ratio for DISH was 1.68, which was even higher than that for diabetes (hazard ratio = 1.25).

**Table 2:** Adjusted hazard ratio of stroke in patients based on age, gender, and comorbidities.

Variables	Adjusted hazard ratio	95% CI	*P* value
**Age**			
50–59	1.00	–	–
60–69	1.89	(1.53–2.33)	< 0.001*
≥ 70	2.54	(2.08–3.1)	< 0.001*
**Gender**			
Female	1.00	–	–
Male	1.12	(0.96–1.3)	0.147
**DISH**			
No	1.00	–	–
Yes	1.68	(1.43–1.98)	< 0.001*
**Hypertension**			
No	1.00	–	–
Yes	2.18	(1.85–2.56)	< 0.001*
**Diabetes mellitus**			
No	1.00	–	–
Yes	1.25	(1.02–1.53)	0.035*
**Hyperlipidemia**			
No	1.00	–	–
Yes	1.16	(0.96–1.42)	0.126
**Chronic kidney disease**			
No	1.00	–	–
Yes	1.31	(0.68–2.54)	0.420

*DISH* diffuse idiopathic skeletal hyperostosis, *CI* confidence interval.

*p < 0.05.

Because many of the risk factors of stroke, such as hypertension, diabetes mellitus, and hyperlipidemia were more prevalent in patients with DISH in our study, further stratified analysis was conducted, and the data are shown in Table 3. The incident rate of stroke was 29.8 per 1000 person-years in DISH patients, and 15.1 per 1000 person-years in patients without DISH. A higher incident rate of stroke was observed in patients who were older, and in those with hypertension, diabetes mellitus, hyperlipidemia, or chronic kidney disease, whether DISH existed or not. Patients with DISH had a 1.68 times greater risk of developing stroke in the overall study population, while the hazard ratios were all above 1 in all of the subgroups. Significantly higher hazard ratios could be observed between the two genders, the 60–70 years and above 70 years age subgroups, and the with and without hypertension subgroups. However, statistical significance could only be established in patients without diabetes mellitus, hyperlipidemia, and chronic kidney disease.

**Table 3:** Stratified analysis of incident rate and hazard ratio of stroke associated with DISH in Cox regression analysis.

	DISH						Crude HR	(95% CI)	*P* value	Adjusted HR	(95% CI)	*P* value
	No (n = 4240)			Yes (n = 1060)								
	Event	Person-years	Incident rate of stroke^a^	Event	Person-years	Incident rate of stroke^a^						
Overall	482	31,835	15.1	222	7452	29.8	1.95	(1.67–2.29)	< 0.001	1.68	(1.43–1.98)	< 0.001
**Age, years**												
50–59	104	12,290	8.5	39	3022	12.9	1.52	(1.05–2.2)	0.026	1.36	(0.94–1.98)	0.102
60–69	165	9436	17.5	77	2402	32.1	1.83	(1.39–2.4)	< 0.001	1.71	(1.3–2.24)	< 0.001
≥ 70	213	10,110	21.1	106	2028	52.3	2.42	(1.92–3.06)	< 0.001	1.77	(1.38–2.26)	< 0.001
**Sex**												
Female	289	18,020	16	111	4364	25.4	1.58	(1.27–1.97)	< 0.001	1.36	(1.09–1.7)	0.007
Male	193	13,814	14	111	3088	36	2.53	(2.01–3.2)	< 0.001	2.18	(1.72–2.77)	< 0.001
**Hypertension**												
No	242	23,896	10.1	95	4414	21.5	2.12	(1.67–2.68)	< 0.001	2.14	(1.68–2.72)	< 0.001
Yes	240	7939	30.2	127	3037	41.8	1.38	(1.11–1.71)	0.004	1.36	(1.09–1.68)	0.006
**Diabetes mellitus**												
No	386	28,657	13.5	184	6338	29	2.14	(1.79–2.55)	< 0.001	1.86	(1.55–2.22)	< 0.001
Yes	96	3178	30.2	38	1114	34.1	1.12	(0.77–1.64)	0.542	1.13	(0.77–1.66)	0.534
**Hyperlipidemia**												
No	382	28,151	13.6	166	5799	28.6	2.09	(1.74–2.51)	< 0.001	1.87	(1.55–2.25)	< 0.001
Yes	100	3684	27.1	56	1652	33.9	1.25	(0.9–1.73)	0.189	1.21	(0.87–1.68)	0.258
**Chronic kidney disease**												
No	477	31,650	15.1	218	7400	29.5	1.94	(1.65–2.28)	< 0.001	1.67	(1.42–1.96)	< 0.001
Yes	5	185	27	4	52	76.8	2.84	(0.76–10.59)	0.120	3.17	(0.76–13.17)	0.112

*DISH* diffuse idiopathic skeletal hyperostosis, *CI* confidence interval, *HR* hazard ratio.

^a^Per 1000 person-years, *p < 0.05.

**Figure 1:**
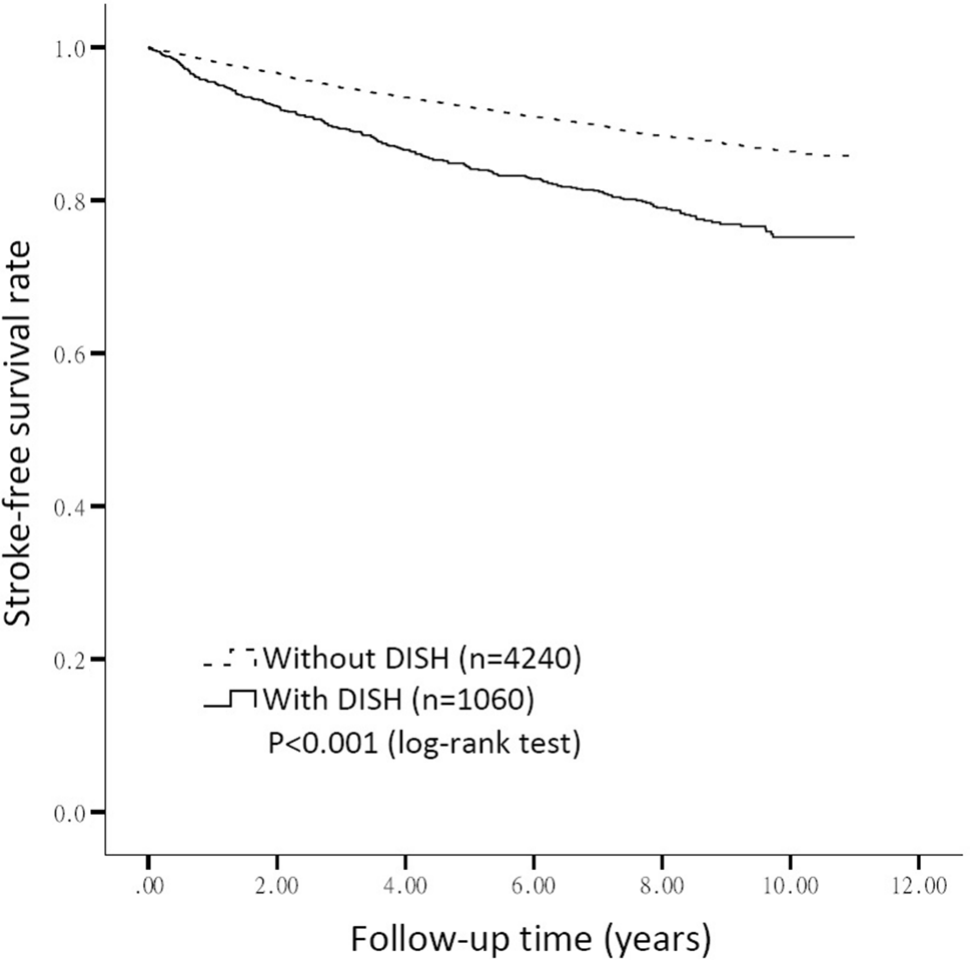
The Kaplan–Meier survival analysis of the stroke-free survival rate in patients with and without diffuse idiopathic skeletal hyperostosis. We used SAS software, version 9.4 (SAS Institute Inc., Cary, NC, USA) for statistical analysis in this study.

Further Kaplan–Meier survival analysis was done to determine the stroke-free survival rate, and the result of the log rank test is shown in Fig. 1. A significantly lower stroke-free survival rate was noted in patients with DISH comparing to those without. (*p* < 0.001).

## Discussion

To our knowledge, this study is the first large-scale population-based investigation to examine the relationship between DISH and the development of stroke. Our results clearly indicate that DISH confers a higher risk of stroke in almost all of the subgroups of patients. In a previous case–control study^19^ recruiting only 45 patients with DISH, a significantly higher incidence of cerebral infarction (*p* = 0.012) and stenosis of major cerebral arteries (*p* = 0.003) were found. Our study further disclosed the hazard ratio of stroke attack through adding the time factor to perform survival analysis. In addition, stratified analysis of different groups of patients further strengthened the evidence of the hazard conferred by DISH.

Metabolic syndrome is considered to be a major risk factor for cardiovascular diseases^21^, and the criteria for diagnosis are the existence of at least three of the following five components: abdominal obesity, hypertriglyceridemia, low high-density lipoprotein cholesterol, hypertension, and high fasting blood glucose. As DISH has been proven to be closely associated with metabolic syndrome^15–17^, our study again provides solid evidence that patients with DISH have a higher prevalence of hypertension, diabetes mellitus, and hyperlipidemia. In the past, higher blood pressure was observed in DISH patients^9,22^. While the mechanism remains unclear, our study also showed a much higher prevalence of hypertension in DISH patients (44%) compared with patients without DISH (27.8%). Our results further revealed a higher hazard ratio of stroke in DISH patients whether they have hypertension or not, suggesting the underlying pathogenesis of stroke in DISH patients may not involve hypertension. However, further study is needed to verify this hypothesis.

An important postulation of the pathogenesis of DISH involves the stimulation of mesenchymal cells to differentiate into chondrocytes and the subsequent endochondral ossification induced by insulin and insulin-like growth factor-1^23,24^. In patients with type 2 diabetes mellitus, hyperinsulinemia is not uncommon because of the elevated insulin resistance in peripheral tissues. Therefore, it can be inferred that a higher risk of subsequent development of DISH exists in patients with diabetes mellitus^22^. In our study, the prevalence of diabetes mellitus was significantly higher in DISH patients (15.2%) compared with patients without DISH (11.5%). While patients with diabetes had a higher risk of developing stroke (hazard ratio = 1.25, *p* = 0.035*), our study revealed DISH itself has a significant hazard ratio of 1.86 (*p* < 0.001*) even in patients without diabetes. The same phenomenon was observed in our patients without hyperlipidemia or chronic kidney disease (hazard ratio = 1.87, *p* < 0.001 and hazard ratio = 1.67, *p* < 0.001, respectively), signifying that DISH may contribute to the development of stroke through a pathway other than metabolic syndrome, which deserves further investigation.

Our study clearly indicated a higher risk of stroke in DISH patients. One of the strengths of this study is the population-based retrospective cohort design, which allowed us to trace the outcome of stroke in every case from the database without loss of follow-up. Furthermore, the large sample size, in contrast to previous observational studies, provides sufficient statistical power in the calculation of the hazard ratio of stroke. However, there were three limitations in this study. First, the diagnosis of stroke and DISH was provided by the NHIRD, and the ICD-9-CM coding may not have been consistent among doctors throughout the country. We tried to avoid this limitation by only enrolling patients with at least three instances of DISH diagnosis during outpatient visits, and stroke was defined only when it was the major inpatient diagnosis during hospitalization. Therefore, the possibility of the under-reporting of radiologist resulting in some DISH cases in the control group should be very low. In addition, DISH is not a frequent temporary diagnosis encountered by outpatient doctors seeing patients with low back pain^1^. While lumbosacral spondylosis (ICD-9-CM 721.3) and herniated lumbar intervertebral disc (ICD-9-CM 722.10) are frequently suspected in the diagnosis and are often over-coded, DISH is often diagnosed only after doctors have viewed the patient’s X-ray. Second, some risk factors for stroke, such as smoking, obesity, sedentary lifestyle, and alcohol consumption, are seldom coded by clinicians, and are thus not provided by the NHIRD. Therefore, we could neither match these risk factors during selection of the subjects in the control cohort nor statistically adjust these risk factors during the Cox regression analysis. However, many of these risk factors can also contribute to hypertension^25^ and diabetes mellitus^26^. By including hypertension and diabetes into the statistical adjustment, these risk factors could be at least partially adjusted for simultaneously. Finally, the database provided by the NHIRD was derived from medical data from Taiwan’s population, and therefore the results may not be generalizable to other populations. Nevertheless, our study findings are still valuable for healthcare workers in Asian countries, reminding them the risk brought by DISH, which is usually considered to be an asymptomatic and harmless disease.

Our study results demonstrate that DISH confers a higher risk of stroke, even in those without hypertension, diabetes, hyperlipidemia, and chronic kidney disease. Our results serve as a reminder to healthcare providers that, in the treatment of patients with DISH, steps should be taken to reduce the incidence of stroke, including more intensive medication control for metabolic syndrome, as well as promotion of a healthier diet, and a less sedentary lifestyle. DISH can be considered to be a warning sign of CVA in the near future, and should never be ignored when encountered incidentally during outpatient visits.

## Methods

### The database

Our study analyzed data from Taiwan’s National Health Insurance Research Database (NHIRD), which contains a subset of anonymous data of one million randomly selected beneficiaries in the National Health Insurance program from 2005 to 2015. The data include each patient’s diagnosis according to the International Classification of Diseases, Ninth revision, Clinical Modification (ICD-9-CM), the date of medical visits, the medicine prescribed, the date of birth, and gender. The National Health Insurance Administration is the single largest medical insurance organization in Taiwan, and about 99.9% of Taiwanese were enrolled in the National Health Insurance program^27^. The data are highly representative of the medical health status of the Taiwanese population, and has been used extensively in public health research since the establishment of the NHIRD^28^. The study was approved and written informed consent from study subjects was not required and waived by the Institutional Review Board of Research Ethics Committee of Taichung Veterans General Hospital, because the NHIRD comprises de-identified data for research purposes (No. CE17178A-3).

### Study design

This was a retrospective cohort study. The study cohort constituted patients with a diagnosis of DISH (ICD-9-CM code 721.6) after X-ray confirmation at least three times during outpatient visits from January 2005 to December 2010. Because DISH has rarely been reported in people less than 50 years old^29,30^, those who were younger than 50 years of age were excluded. Furthermore, patients with a diagnosis of stroke (ICD-9-CM code 430–438) before the date of DISH diagnosis were also excluded to avoid confounding the analysis of the causal relationship. As a result, a total of 1060 patients with DISH were selected as the study cohort.

**Figure 2:**
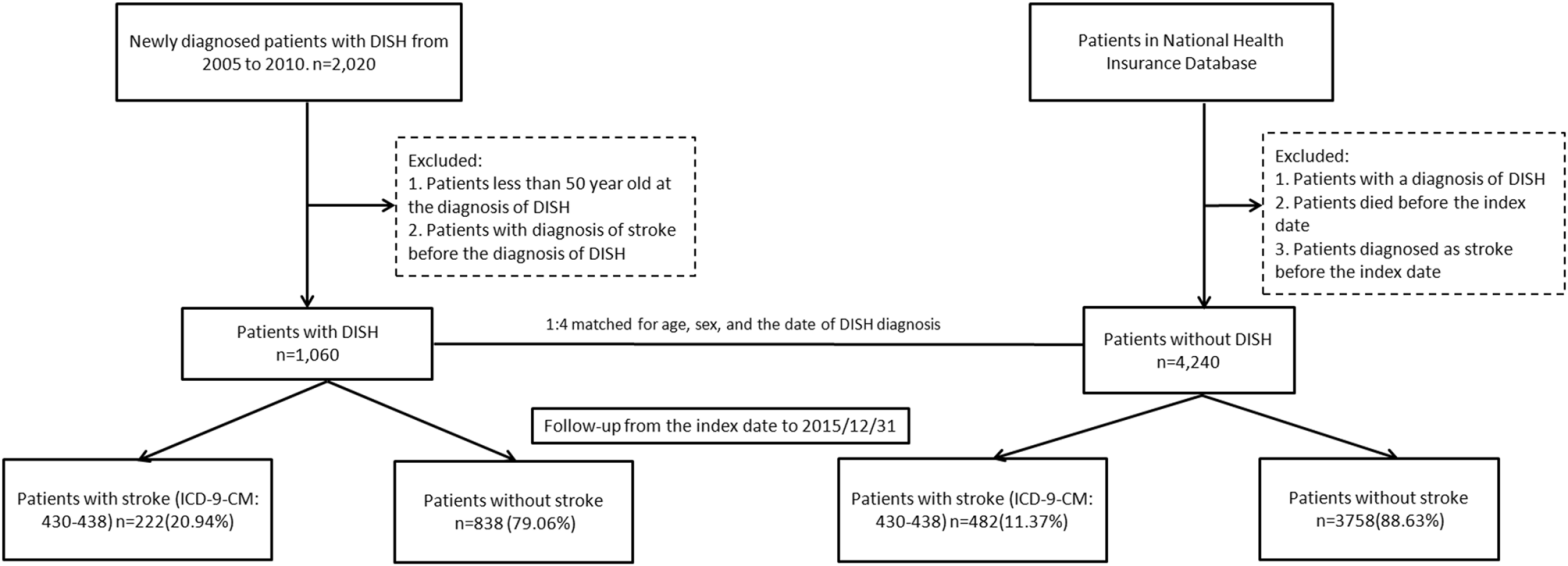
The flowchart of enrollment of study subjects.

Subjects in the comparison cohort were then selected from the remaining patients in the database and statistically matched for age, sex, and the date of DISH diagnosis with a ratio of 1:4. Those with a diagnosis of stroke before the index date of the study were also excluded, just as with the study group. As a consequence, the comparison cohort comprised 4240 patients without DISH. All subjects in both the study and the comparison cohort were followed until the end of 2015 to trace the development of stroke. In order to avoid mistaken diagnosis, only those with ICD-9-CM code 430–438 as the major inpatient diagnosis during hospitalization were defined as occurrence of stroke. In addition, medical comorbidities including hypertension (ICD-9-CM code 401–405), diabetes mellitus (DM) (ICD-9-CM code 250), hyperlipidemia (ICD-9-CM code 272), and chronic kidney disease (CKD) (ICD-9-CM code 585) were evaluated in order to conduct statistical adjustment as they are risk factors of stroke. The criteria for confirming diagnosis of these diseases were the same as those for DISH, namely, at least three times of established diagnoses during outpatient visits. The flowchart of our study design is shown in Fig. 2.

### Statistical analysis

We used SAS software, version 9.4 (SAS Institute Inc., Cary, NC, USA) for statistical analysis in this study (https://www.sas.com/en_us/legal/editorial-guidelines.html). Chi-squared test or Fisher's Exact Test were conducted to examine the distributions of age, sex, and medical comorbidities, including stroke, hypertension, DM, hyperlipidemia, and CKD between those with and without DISH. Next, Cox regression analysis was performed to compute the hazard ratio of stroke according to the subjects’ age, gender, and the presence of DISH, hypertension, DM, hyperlipidemia, and CKD. Statistical adjustment was performed, which included all of the variables. Further stratified analysis based on gender, different comorbidites, and ages from 50 to 60, 60 to 70, and over 70 years was also performed to determine the hazard of DISH in different groups. Finally, Kaplan–Meier survival analysis was performed to determine the stroke-free survival rate of patients with and without DISH. A *p* < 0.05 was considered to be statistically significant in this study.

## Data Availability

The datasets analyzed during the current study are available from the corresponding author on reasonable request. All methods were carried out in accordance with relevant guidelines and regulations.
